# LiV_3_O_8_/Polytriphenylamine Composites with Enhanced Electrochemical Performances as Cathode Materials for Rechargeable Lithium Batteries

**DOI:** 10.3390/ma10040344

**Published:** 2017-03-26

**Authors:** Wenjuan Li, Limin Zhu, Ziheng Yu, Lingling Xie, Xiaoyu Cao

**Affiliations:** 1 School of Chemistry and Chemical Engineering, Henan University of Technology, Zhengzhou 450001, China; liwenjuan_1991@163.com (W.L.); linglingxie51@163.com (L.X.); 2 Key Laboratory of High Specific Energy Materials for Electrochemical Power Sources of Zhengzhou City, Henan University of Technology, Zhengzhou 450001, China; 3 School of Pharmacy, China Pharmaceutical University, Nanjing 211196, China; zihengyu@126.com

**Keywords:** LiV_3_O_8_/polytriphenylamine composites, cathode materials, in situ chemical polymerization method, rechargeable lithium batteries, electrochemical performances

## Abstract

LiV_3_O_8_/polytriphenylamine composites are synthesized by a chemical oxidative polymerization process and applied as cathode materials for rechargeable lithium batteries (RLB). The structure, morphology, and electrochemical performances of the composites are characterized by X-ray diffraction, scanning electron microscopy, transmission electron microscopy, galvanostatic discharge/charge tests, and electrochemical impedance spectroscopy. It was found that the polytriphenylamine particles were composited with LiV_3_O_8_ nanorods which acted as a protective barrier against the side reaction of LiV_3_O_8_, as well as a conductive network to reduce the reaction resistance among the LiV_3_O_8_ particles. Among the LiV_3_O_8_/polytriphenylamine composites, the 17 wt % LVO/PTPAn composite showed the largest *d*_100_ spacing. The electrochemical results showed that the 17 wt % LVO/PTPAn composite maintained a discharge capacity of 271 mAh·g^−1^ at a current density of 60 mA·g^−1^, as well as maintaining 236 mAh·g^−1^ at 240 mA·g^−1^ after 50 cycles, while the bare LiV_3_O_8_ sample retained only 169 and 148 mAh·g^−1^, respectively. Electrochemical impedance spectra (EIS) results implied that the 17 wt % LVO/PTPAn composite demonstrated a decreased charge transfer resistance and increased Li^+^ ion diffusion ability, therefore manifesting better rate capability and cycling performance compared to the bare LiV_3_O_8_ sample.

## 1. Introduction

Lithium trivanadate (LiV_3_O_8_, LVO) has received considerable attention as a promising cathode material based on its remarkable electrochemical performance, such as high theoretical capacity [1,2,3,4,5], high working voltage, and low cost [6,7]. It is well-know that the electrochemical performances of LVO depends highly upon preparation methods and post-processing techniques [8,9]. Therefore, several synthetic routes have been developed to further enhance the electrochemical performance of LVO, such as microwave synthesis [2], sol-gel synthesis [10,11,12,13,14], spray pyrolysis synthesis [15,16], hydrothermal synthesis [2,17], and solid-state reactions [18,19]. However, until now, bare LVO still suffers from low capacity retention and poor rate performance on account of the phase transformation and slow kinetics during the charge-discharge process [20,21,22], which hinders its practical applications.

Recently, the use of polymers or inorganic-polymer composites as electrodes has attracted interest, as it shows good capacity for retention, and long cycle life [23,24]. Coating or compositing with redox-active polymers [25,26,27,28,29] is an effective strategy to ameliorate the electrochemical performances of LVO, due to their high electronic conductivities and quick electrochemical kinetics [30]. Moreover, redox-active polymers can confine the dissolution of LVO into electrolyte and parasite reactions between the LVO surface and the electrolyte [31]. Among the various redox-active polymers, polytriphenylamine (PTPAn) [32] has excellent cycleability and rate capability, owing to the conductive polyparaphenylene backbone and electroactive polyaniline structure model that have been developed as cathode materials for RLB. However, as far as we know, the synthesis and application of LVO/PTPAn composites as the cathode materials for RLB have not yet been reported. Hence, in this work, we elaborated the synthesis of PTPAn/LVO nanocomposites via in situ oxidative polymerization. The electrochemical performances of LVO/PTPAn nanocomposites were studied in detail and compared with the bare LVO sample, showing the enhanced rechargeable capacities and cycling performances.

## 2. Experimental

The bare LVO sample was synthesized by a rheological phase reaction technique [33]. First, the stoichiometrically weighed raw materials (LiOH·H_2_O, NH_4_VO_3_, and C_6_H_8_O_7_·6H_2_O) were ground thoroughly in an agate mortar, then the mixture was transferred into a cylindrical Teflon-lined autoclave, and the mixture was stirred mechanically, with dropwise addition of deionized water. When the solid-liquid rheological body presented its intrinsic appearance, the cylindrical Teflon-lined autoclave was placed in an air oven at 80 °C for eight hours. After cooling to 25 °C, the materials were dried again at 100 °C for 12 h. The precursor materials were then calcined in an aerated muffle furnace at 350 °C for 10 h to obtain the bare LVO sample.

The LVO/PTPAn composites were prepared by in situ chemical polymerization of triphenylamine (TPA) using chloroform (CHCl_3_) as a solvent and ferric trichloride (FeCl_3_) as an oxidant. LVO (0.2 g), TPA monomer (0.04 g) (0.162 mmol, 20 wt % PTPAn content) and CHCl_3_ (50 mL) were added into a flask and stirred for 10 min in N_2_, and then 0.648 mmol FeCl_3_ oxidant was divided into four portions and added within 1 h. The mixture was constantly stirred at 50 °C for six hours. Finally, the solution was poured into methanol to precipitate, then washed with deionized water and dried at 80 °C overnight to acquire the LVO/PTPAn composite with a content of 20 wt % PTPAn. The preparation of other LVO/PTPAn composites (30 and 40 wt % PTPAn content) was similar to the above route, except for the amount of TPA monomer.

To determine the content of PTPAn in the composites, thermogravimetric analysis (TGA) was implemented via a Setaram 92 instrument (Setaram Instrumontation, Lyons, France). Power X-ray diffraction (XRD) measurement was carried out through Rigaku MiniFlex 600 diffractometer (Rigaku Co., Tokyo, Japan) with Cu Kα radiation. The morphological features of the specimen were examined by scanning electron microscopy (SEM, Quanta 250 FEG, FEI, Hillsboro, OR, USA) and transmission electron microscopy (TEM, HT7700, HITACHI, Tokyo, Japan). The Fourier transform infrared (FT-IR) spectra of samples were verified on a Nicolet Avatar 360 (Nicolet Instrument Co., Madison, WI, USA) with KBr pellets.

The cathode film was fabricated by 70 wt % as a prepared composite, with 20 wt % acetylene black and 10 wt % polytetrafluoroethylene (PTFE) microemulsion (60 wt %) added into the paste, then rolled into a sheet and dried in an oven. The sheet was then pressed on an aluminum net. The discharge/charge test was accomplished using 2016 type coin cells. The cells were composed of a cathode, a Li metal anode, a commercial polyethylene separator and 1 mol·dm^−3^ LiPF_6_ in ethylene carbonate, ethyl methyl carbonate and dimethyl carbonate (EC/EMC/DMC) (1:1:1, *v*/*v*/*v*, provided by Zhangjiagang Guotai-Huarong New Chemical Materials Co., Ltd., Suzhou, China) electrolyte and assembled in an argon-filled glove box (JMS-3, Nanjing Jiumen Automation technology Co., Ltd., Nanjing, China). The assembled cells were galvanostatically discharged and charged on a Land battery tester (CT2001A, Wuhan Land Electronic Technology Co., Ltd. Wuhan, China). Cyclic voltammetry (CV) and EIS were performed on a CHI 660D electrochemical workstation (Shanghai ChenHua Instruments Co., Shanghai, China). The amplitude of EIS signal was ±5 mV and the frequency ranged from 100 kHz to 10 mHz under the open-circuit condition. The recorded specific capacities of LVO/PTPAn composites are rely on the mass of the LVO/PTPAn samples.

## 3. Results and Discussion

**Figure 1:**
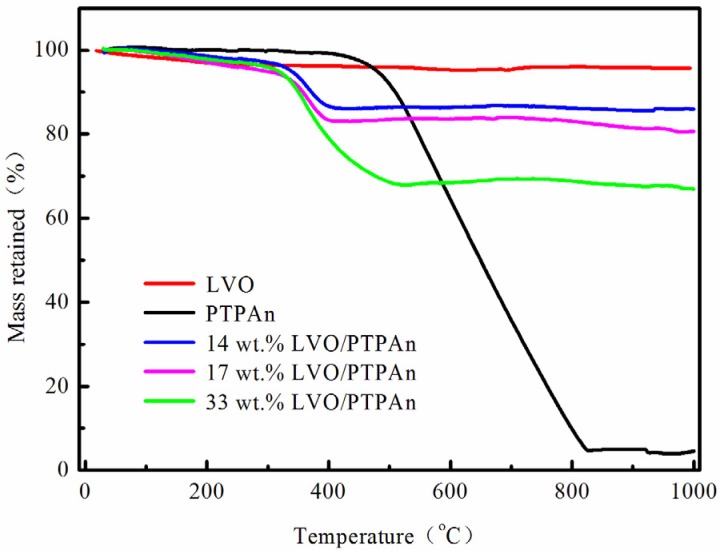
Thermogravimetric analysis (TGA) curves of polytriphenylamine (PTPAn) powder, the bare lithium trivanadate (LVO) sample, and LVO/PTPAn composites.

To determine the content of PTPAn in the composites, TGA was implemented in an aerated environment with a heating rate of 5 °C·min^−1^. As shown in Figure 1, there was almost no weight loss for the bare LVO sample until 1000 °C; however PTPAn began to decompose at about 450 °C, and fully broke down at around 800 °C. Hence, we can calculate the actual amount of PTPAn in the LVO/PTPAn composites with the content of 20 wt %, 30 wt %, and 40 wt % are 14 wt %, 17 wt %, and 33 wt % according to TG curves, respectively. In the following, these three LVO/PTPAn composites are called 14 wt % LVO/PTPAn, 17 wt % LVO/PTPAn, and 33 wt % LVO/PTPAn, respectively.

**Figure 2:**
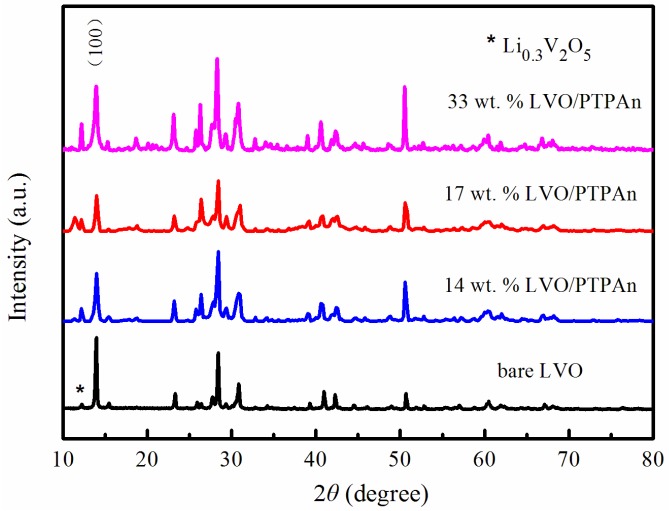
Power X-ray diffraction (XRD) patterns of the bare LVO sample and LVO/PTPAn composites.

XRD patterns of the bare LVO sample and LVO/PTPAn composites are shown in Figure 2. The bare LVO sample showed good crystal structure, and was a potential monoclinic crystalline LVO phase (JCPDS 72-1193, space group: *P2*1/*m*) [34,35], and there was a redundant peak assigned to Li_0.3_V_2_O_5_, which can be usually be observed in the LVO [36,37,38]. The LVO/PTPAn composites could also be ascribed to the LVO structure, while the PTPAn phase was not observed, which may be attributed to the amorphous structure of the polymer. Importantly, the 17 wt % LVO/PTPAn composite showed the weakest X-ray diffraction peak intensity, which suggests that this composite had lower crystallization levels [18,39]. There was a slight shift of the (100) diffraction peak in contrast to the normative patterns of LVO, signifying that PTPAn may be intercalated into the interlayer of LVO crystals during the synthesis process, according to previous reported works [40,41]. Using the Bragg equation of 2*d*_100_sin*θ*_100_ = *nλ*, the lattice plane spacing (*d*_100_) of the crystal was calculated and recorded in Table 1. As shown in Table 1, the *d*_100_ spacing first increased, and then subsequently decreased. A maximum *d*_100_ spacing of 6.5727 Å was reached for the 17 wt % LVO/PTPAn composite. It is well known that a larger *d*_100_ spacing facilitates Li^+^ ions mobility and distribution in the framework of electroactive host material, which suggests that the 17 wt % LVO/PTPAn composite may demonstrate the excellent electrochemical performances.

**Figure 3:**
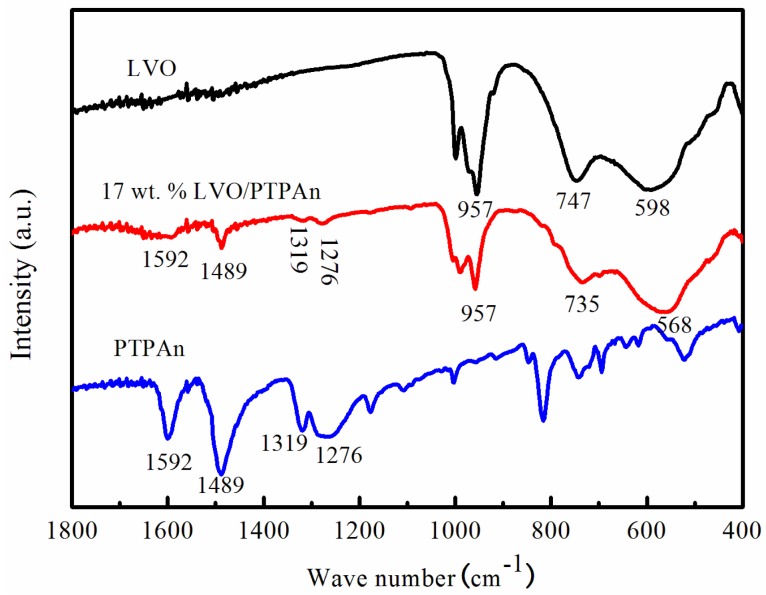
The FT-IR spectra of the bare LVO sample, the PTPAn sample and the 17 wt % LVO/PTPAn composite.

The FT-IR spectra of the bare LVO sample, PTPAn polymer and 17 wt % LVO/PTPAn composite are displayed in Figure 3. The FT-IR spectrum of the bare LVO sample showed three strong infrared absorption characteristic peaks at 957, 746, and 598 cm^−1^, which are characteristic of the V=O, the symmetric V-O-V, and the asymmetric V-O-V stretching vibrations, respectively [42,43,44]. Moreover, the FT-IR spectrum of the 17 wt % LVO/PTPAn composite showed new absorption bands at 1592, 1489, 1319, and 1276 cm^−1^, which are attributed to C=C stretching due to the quinoid structures formed, C–C stretching, C–H bending, and C–H out-of-plane vibrations of the PTPAn [32]. The IR features implied that the LVO/PTPAn composite was successfully formed in this study.

**Figure 4:**
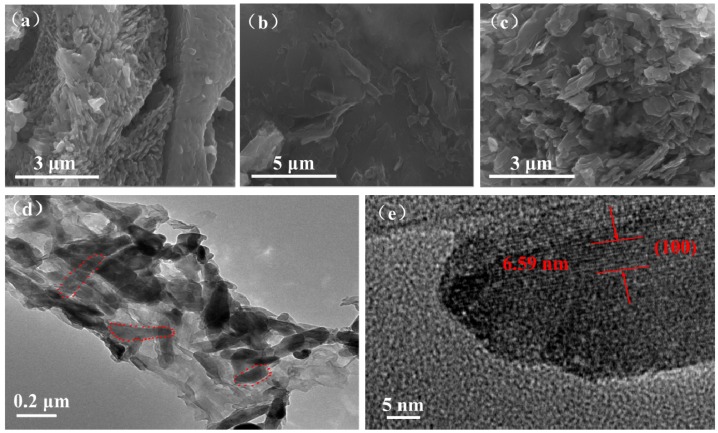
(**a**) Scanning electron microscope (SEM) micrograph of the bare LVO sample; (**b**) PTPAn polymer; (**c**) 17 wt. %LVO/PTPAn composite; (**d**) TEM micrograph of the 17 wt % LVO/PTPAn composite; (**e**) high-resolution transmission electron microscope (HRTEM) micrograph of the 17 wt % LVO/PTPAn composite.

The SEM and TEM micrographs of the bare LVO sample, PTPAn polymer, and the 17 wt % LVO/PTPAn composite are shown in Figure 4. The bare LVO sample showed a rough surface which exists in the shape of nanorods with diameters of 100–200 nm, and the PTPAn appeared flaky with a coralloid surface, while the 17 wt % LVO/PTPAn composite showed a smooth surface, and the diameters of nanorods increased to around 200–300 nm, which indicated that the surface of LVO is coated by the PTPAn. The TEM image revealed that the surfaces of the LVO nanorods were coated with PTPAn. The high-resolution TEM image (Figure 4e) of a selected nanofiber showed clear crystal lattices with a d-spacing of 0.659 nm, agreeing with the interplanar distance of the (100) plane of the LVO, which coincides with the XRD results shown in Table 1.

**Figure 5:**
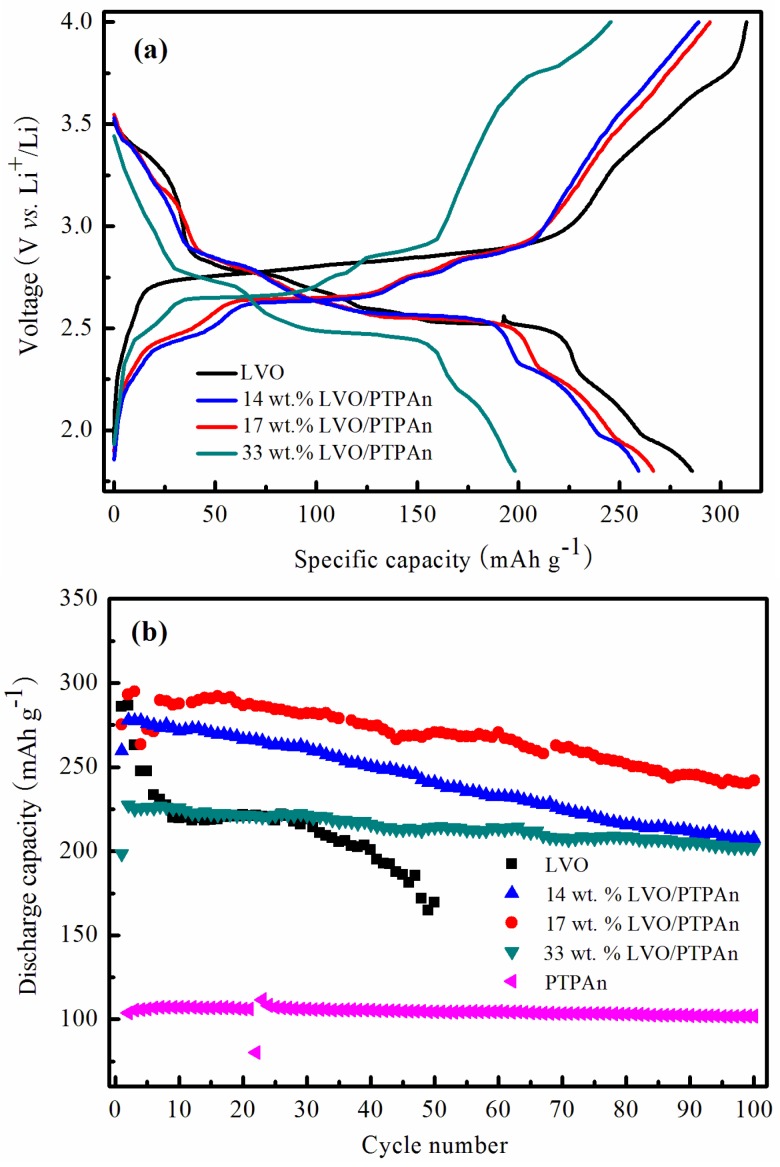

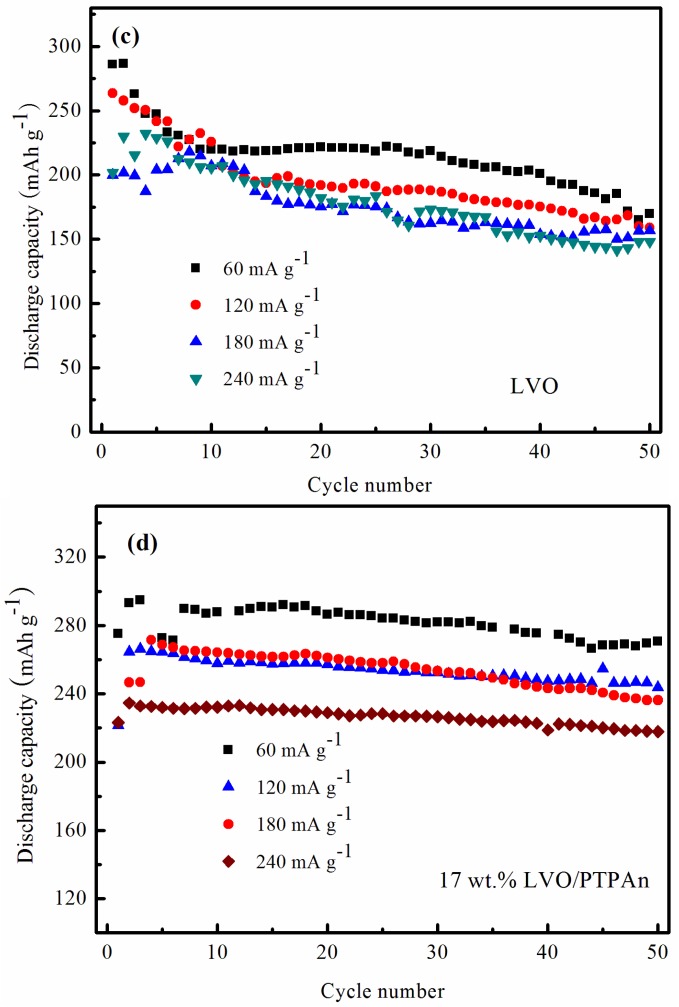
(**a**) Discharge/charge curves for the first cycle of the bare LVO sample and LVO/PTPAn composites; (**b**) cycle life of the bare LVO sample and LVO/PTPAn composites at the current rate of 60 mA·g^−1^; **(c)** discharge capacities of the bare LVO sample at different current rates; (**d**) discharge capacities of 17 wt % LVO/PTPAn composite at the different current rates in the range of 1.8–4.0 V.

The charge-discharge behaviors for the initial cycle of the bare LVO sample and LVO/PTPAn composites between 1.8 and 4.0 V (vs. Li^+^/Li) at the current density of 60 mA·g^−1^ are presented in Figure 5a. The bare LVO sample showed several plateaus during the discharge/charge process, with a discharge capacity of 286 mAh·g^−1^. However, the LVO/PTPAn composites showed more noticeable plateaus and a reversible capacity of 260 mAh·g^−1^ for the 14 wt % LVO/PTPAn composite, 267 mAh·g^−1^ for the 17 wt % LVO/PTPAn composite, and 198 mAh·g^−1^ for the 33 wt % LVO/PTPAn composite. It is noteworthy that the bare LVO showed the highest initial discharge capacity, however, the 17 wt % LVO/PTPAn composite showed symmetrical charge and discharge potential plateaus, indicating a lower electrochemical polarization. The results also showed that the specific discharge capacities of the LVO/PTPAn composites increased first and then decreased with increased PTPAn content in the composites, because the 17 wt % LVO/PTPAn composite showed the largest *d*_100_ spacing listed in Table 1. Three discharge plateaus were seen at about 2.82, 2.55, and 2.24 V in the discharge curve of the 17 wt % LVO/PTPAn composite. The first plateau of discharge curves at about 2.82 V agreed with the Li^+^ ion insertion in the tetrahedral sites. The second plateau of discharge curves at around 2.55 V was attributed to the phase transformation of LiV_3_O_8_ to Li_4_V_3_O_8_ [36,45,46], and the last plateau at around 2.24 V represented single-phase transition of the Li_4_V_3_O_8_ [47,48]. 

Figure 5b displays the cycling performance of the bare LVO sample, PTPAn polymer and LVO/PTPAn composites at the current rate of 60 mA·g^−1^. The discharge capacities of the 14 wt % LVO/PTPAn, 17 wt % LVO/PTPAn, and 33 wt % LVO/PTPAn composites increased to 278, 293 and 227 mAh·g^−1^ at the second cycle, then became steady and remained as high as 208, 242, and 202 mAh·g^−1^ at the 100th cycle. The first discharge capacities of the LVO/PTPAn composites were below the succeeding cycles, which could be attributed to the activation of PTPAn during the initial charge-discharge process. However, the first discharge capacity of the bare LVO sample was 286 mAh·g^−1^, this decreased sharply in the successive cycles and dropped to 169 mAh·g^−1^ after 50 cycles. It was observed that the 17 wt % LVO/PTPAn composite showed the best electrochemical performance, which was attributed to the protection given by the additional polymer layer on the LVO sample, this would decrease harmful side reactions between LVO particles and electrolytes, and the dissolved LVO.

The cycling performances of the bare LVO sample and the 17 wt % LVO/PTPAn composite at the different current rates are shown in Figure 5c,d. The bare LVO sample supplied initial discharge capacities of 286, 263, 199, and 201 mAh·g^−1^ at the current rates of 60, 120, 180 and 240 mA·g^−1^ respectively, and remained at 169, 158, 156 and 148 mAh·g^−1^ (59.1%, 60%, 78%, and 74% of the first capacity) at the 50th cycle, respectively. On the other hand, the 17 wt % LVO/PTPAn composite provided initial capacities of 275, 222, 223 and 247 mAh·g^−1^ at the same current rates, and kept 271, 244, 218, and 236 mA·hg^−1^ (98.5%, 110%, 97.8%, and 95.5% of the first capacity) at the 50th cycle, respectively. This data showed that the 17 wt % LVO/PTPAn composite has a favorable rate performance and structural stability, even if operated at a high current rate, and can act as a high-performance cathode material for RLB.

**Figure 6:**
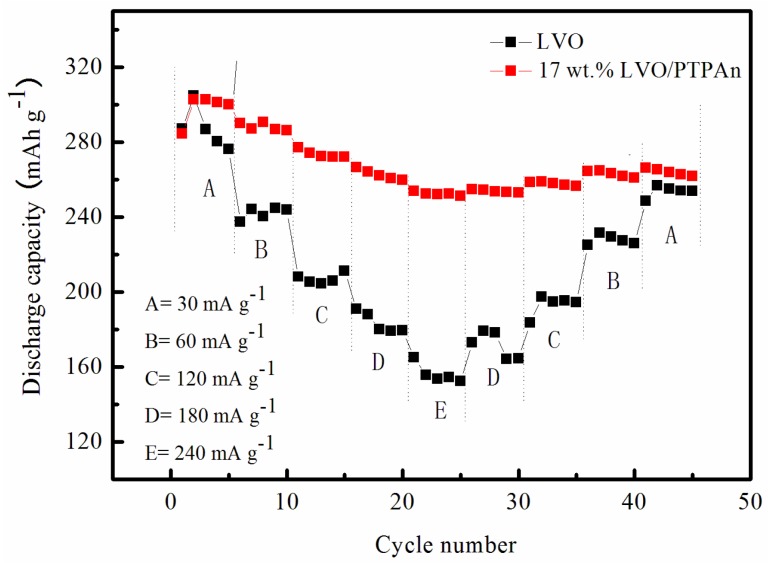
Plots of discharge capacity of the bare LVO sample and 17 wt % PTPAn/LVO composite at different current rates ranging from 30 mA·g^−1^ to 240 mA·g^−1^.

As part of the evaluation of the electrochemical performance of the 17 wt % LVO/PTPAn composite, the symmetric stepped rate capability at the various current rates are given in Figure 6. When the current was increased from 30 mA·g^−1^ to 120 mA·g^−1^, the 17 wt % LVO/PTPAn composite still provided a capacity of around 263 mAh·g^−1^. Even with a higher current rate of 240 mA·g^−1^, the 17 wt % LVO/PTPAn composite remained at a capacity of 244 mAh·g^−1^. Additionally, the rate capability of the 17 wt % LVO/PTPAn composite was much better than the bare LVO sample. Even after 60 cycles, the current rates were restored to 30 mA·g^−1^. The 17 wt % LVO/PTPAn composite was able to recover to near-primary capacity, showing excellent cycling stability. As stated above, we found that 17 wt % LVO/PTPAn composite exhibited better cycling stability and rate capability, which may be explained by the following: First, in comparison with the bare LVO and other LVO/PTPAn composites, 17 wt % LVO/PTPAn composite has the largest *d*_100_-spacing, promoting quicker movement of Li^+^ ions in the host material. Second, PTPAn coating on the surface of LVO may restrain the dissolution of LVO to improve the cycle stability of the LVO. Third, PTPAn composited with LVO may enhance the electrical conductivity of the LVO.

**Figure 7:**
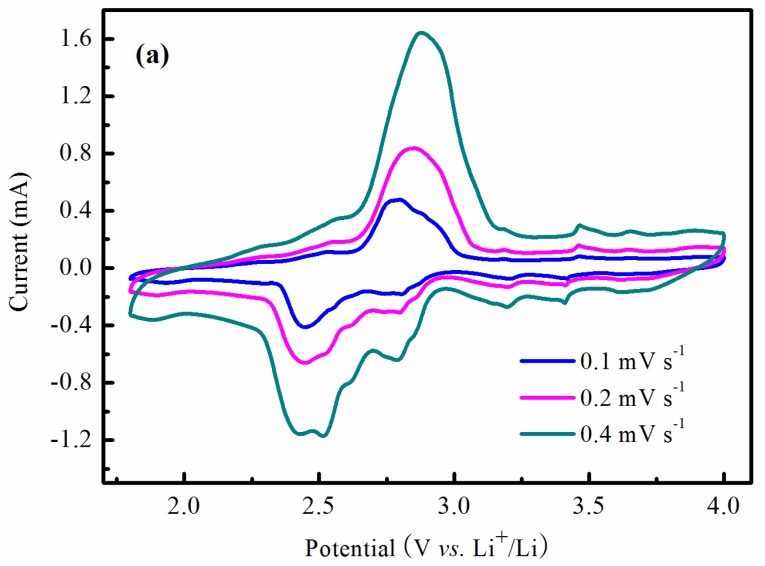

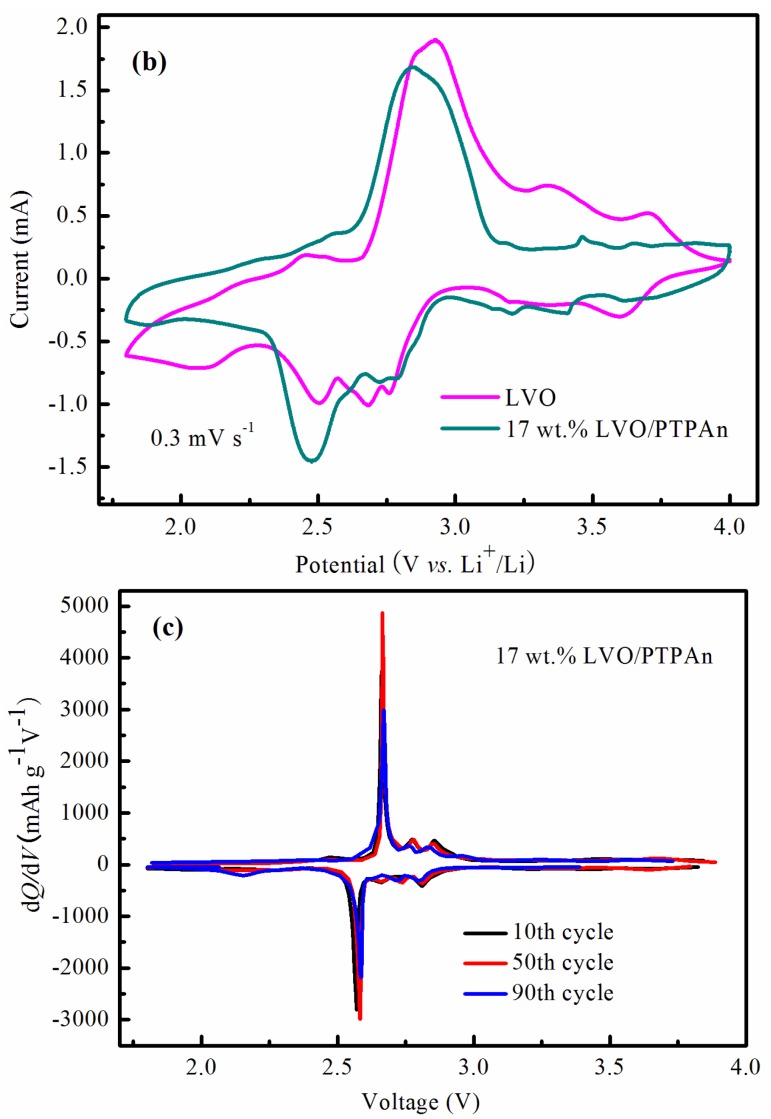
(**a**) The second cyclic voltammetry (CV) curves of the 17 wt % LVO/PTPAn composite at different scan rates in a potential range of 1.8–4.0 V; (**b**) the second CV curves of the bare LVO sample and 17 wt % LVO/PTPAn composite at the scan rate of 0.3 mV·s^−1^; (**c**) the differential capacity curves of 17 wt % LVO/PTPAn composite on the 10th, 50th and 90th cycle.

The CV features of the 17 wt % LVO/PTPAn composite at various scan rates are shown in Figure 7a. As depicted from CV, the height and area of the redox peaks increased with increasing of the scan rate, indicating that the redox process of LVO/PTPAn composite is diffusion-limited in the intercalation/deintercalation processes of Li^+^ ions. The CV of the bare LVO and 17 wt % LVO/PTPAn composite at the second cycle is shown in Figure 7b. Four anodic peaks at 3.71, 3.34, 2.93, and 2.46 V and five cathodic peaks at 2.1, 2.51, 2.68, 2.76, and 3.61 V were observed for the bare LVO sample, which were attributed to the Li^+^ ion insertion/extraction reactions and the phase transformation in Figure 5a. However, only one main broad oxidation peak at 2.82 V and two reduction peaks at 2.47 and 2.76 V were discovered after introduction of PTPAn in the LVO sample. CV results demonstrated that the amount of phase transitions undergone during Li^+^ ions intercalation/deintercalation for LVO/PTPAn composite was decreased, and accordingly, the electrochemical reaction become more reversible due to the suppressed phase transition, thus improving the electrochemical performances of LVO/PTPAn composite. In order to further prove the structural stability of 17 wt % LVO/PTPAn composite, the differential capacity curves on the 10th, 50th, and 90th cycle were compared (Figure 7c). It was observed that the differential capacity curves maintained similar shapes and consistent redox peak positions at different cycles, demonstrating a stable structure for the composite.

**Figure 8:**
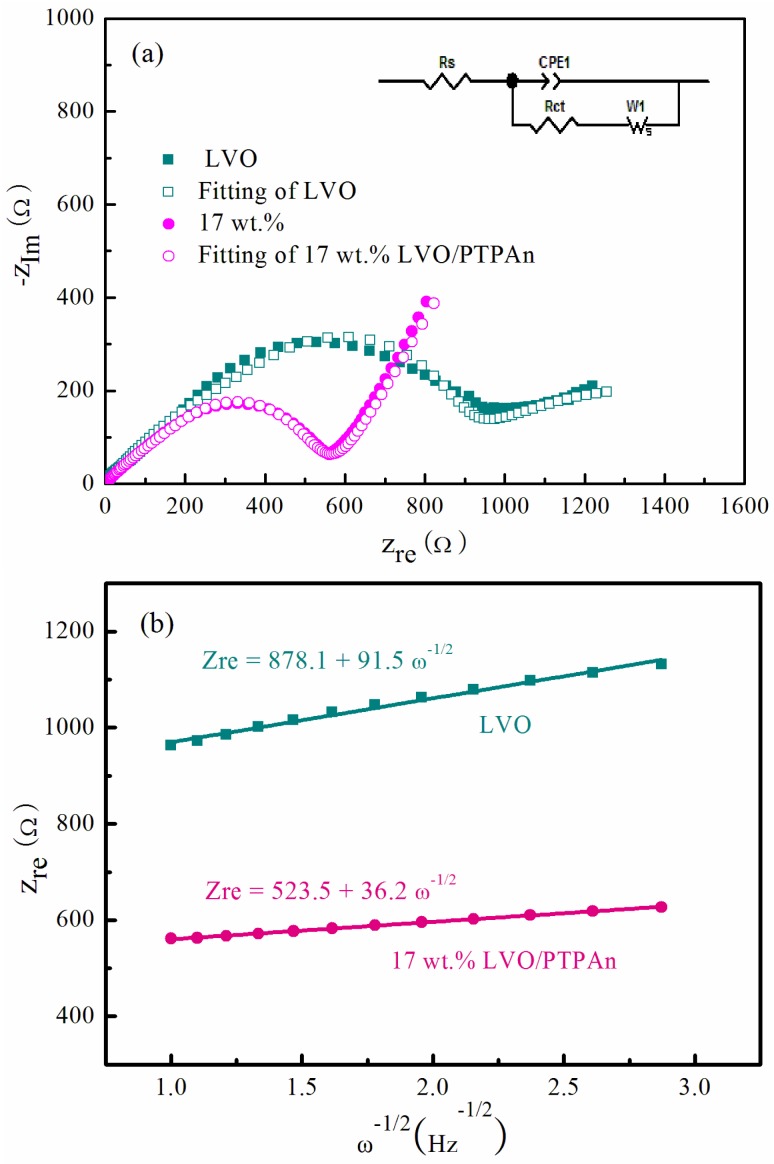
(**a**) Electrochemical impedance spectra (EIS) of the bare LVO sample and the 17 wt % LVO/PTPAn composite under open-circuit conditions; (**b**) and the relationship curves between *Z*_re_ and *ω*^−1/2^ in the low frequency range.

To better understand the reasons for the electrochemical performance of the 17 wt % LVO/PTPAn composite, EIS of the bare LVO sample and the 17 wt % LVO/PTPAn composite were measured and compared for electrochemical resistance, with the results shown in Figure 8. The EIS showed a semicircle in the high frequency region, and a slop line at the low frequency, which were ascribed to the charge-transfer resistance (*R*_ct_) and the Li^+^ diffusion resistance in the electrodes (*Z*_w_), respectively (Figure 8a). The fitting *R*_ct_ of the 17 wt % LVO/PTPAn composite was 425 Ω, which was much less than the LVO sample (767 Ω). It was evident that the PTPAn coating provided both an electronic and Li^+^ ion pathway among the LVO particles, which significantly improved the charge and ionic transfer, consequently elevating the electrochemical performance of LVO sample. Figure 8b shows the line of fit for *Z*_re_ vs. *ω*^−1/2^, from which the slope (σ_ω_, Ω·s^−1/2^) could be attained, and the diffusion coefficient value (*D*) was calculated by the following equation [49]:

*D* = 0.5(*RT*/*An*^2^*F*^2^*σ_ω_C*)^2^ (1)

where *R* is the gas constant (*R* = 8.314 J·K^−1^·mol^−1^), *T* is the temperature (*T* = 298 K), *A* is the effective contact area between the electrode and the electrolyte (*A* = π × 1 cm^2^/4 = 0.7854 cm^2^), *n* is the number of electrons transferred per mole of the active material involved in the electrode reaction (*n* = 3), *F* is Faraday’s constant (*F* = 96500 C·mol^−1^), and *C* is the concentration of the Li^+^ ion in the cathode calculated based on the crystallographic cell parameter of LVO (*n*_Li_^+^ = (3/6.02 × 10^23^) mol, *V* = 275.5 Å^3^, *C = n*_Li_^+^/*V* = 1.808 × 10^4^ mol·m^−3^). The *D*_Li_^+^ value of the bare LVO (σ_ω_ = 91.5 Ω·s^−1/2^) and the 17 wt % LVO/PTPAn composite (σ_ω_ = 36.2 Ω·s^−1/2^) was calculated to be 2.58 × 10^−16^ and 1.65 × 10^−15^ cm^2^·s^−1^, respectively, signifying the quicker Li^+^ ions diffusion ability of LVO/PTPAn composite.

## 4. Conclusions

LVO/PTPAn composites were prepared via an in situ oxidative polymerization route. Compared to the bare LVO sample, the 17 wt % LVO/PTPAn composite had the largest *d*_100_ spacing and exhibited the highest rechargeable capacity, better cycling behavior, and appropriate rate performance. The results revealed that the 17 wt % LVO/PTPAn composite provided a second reversible capacity of 293 mAh·g^−1^ and remained as high as 242 mAh·g^−1^ at the 100th cycle, at the current rate of 60 mA·g^−1^. Even at the high current density of 240 mA·g^−1^, the 17 wt % LVO/PTPAn composite gave an initial capacity of 247 mAh·g^−1^ and maintained 236 mAh·g^−1^ up to 50 cycles, which was attributed to low charge transfer resistance, quick Li^+^ ions diffusion ability, and stable structural characteristics. It was suggested that PTPAn can act both as a conductor to promote the transport of electrons and Li^+^ ions among LVO particles, and as a coating to improve structural stability. These results confirm that LVO composited with proper polymer could be an attractive means for building effective structures for high-performance RLB.

## Figures and Tables

**Table 1:** The *d*_100_ spacing of the as-prepared bare LVO sample and LVO/PTPAn composites.

**Samples**	**Bare LVO**	**14 wt % LVO/PTPAn**	**17 wt % LVO/PTPAn**	**33 wt % LVO/PTPAn**
***d*_100_-spacing (Å)**	6.4361	6.5284	6.5727	6.3449
